# Might female patients benefit more from bariatric surgery with respect to inflammation

**DOI:** 10.3389/fsurg.2022.890116

**Published:** 2022-08-08

**Authors:** Qing Zhou, Pingping Yan, Haiming Shi, Ping Yan

**Affiliations:** Department of Cardiology, Huashan Hospital, Fudan University, Shanghai, China

**Keywords:** bariatric surgery, neutrophil-to-lymphocyte ratio, C-reactive protein, myocardial performance index, global longitudinal strain

## Abstract

**Background:**

Bariatric surgery is an effective method for severe obesity and its related comorbidities, in which inflammation plays a crucial role. The aim of this study was to investigate the changes of Neutrophil-to-lymphocyte ratio (NLR) and C-reactive protein (CRP) in patients undergoing laparoscopic sleeve gastrectomy (LSG) and to explore the related factors including gender.

**Methods:**

We retrospectively included 72 patients undergoing LSG in our hospital from 2017 to 2020. Clinical information, laboratory investigations as well as parameters derived from traditional and 2D strain echocardiography were collected. Univariate logistic model was used in myocardial performance index (MPI) and *E*/*E*′ analysis. Univariate and Multivariate logistic model were used in NLR analysis.

**Results:**

At baseline, all patients had normal left ventricular ejection fraction (LVEF). The myocardial performance index (MPI) (OR = 1.218 (95%CI 1.040, 1.426); *p* = 0.0142) and *E*/*E*′ (OR = 1.364 (95%CI 1.124, 1.655); *p* = 0.0017) were independently associated with CRP. LSG led to a significant decrease in inflammatory markers (NLR, 2.4 ± 1.59 vs.1.7 ± 0.86; CRP, 5.6 ± 3.17 vs. 2.1 ± 2.35 mg/L, respectively, both *p* < 0.001),which was more in NLR among female than male (OR = 3.14 (95%CI 1.112, 8.870); *p* = 0.031).

**Conclusions:**

The present study indicated a significant correlation between subclinical cardiac dysfunction and CRP among obese patients. Furthermore, female patients might benefit more from bariatric surgery on inflammation.

## Introduction

Obesity is one of worldwide health problems, that has nearly tripled since 1975. More than 1.9 billion adults and 39 million children under the age of 5 were overweight or obese in 2020 (1). It has also become an epidemic in China in the latest three decades (2). Overweight and obesity are independent risk factors for hypertension, cardiovascular disease, diabetes, steatohepatitis and even cancer (3). Inflammation is considered to be involved in the occurrence and the development of both obesity and obesity-associated diseases (4).

Laparoscopic sleeve gastrectomy (LSG), one of the most commonly used procedures, is considered as an effective therapeutic option for morbidly obese patients (5). Previous studies indicated LSG could significantly reduced the serum C-reactive protein (CRP) level (6). As a traditional inflammatory marker, CRP is a simple and reliable indicator and has been widely used to assess the inflammation burden (7). The baseline level of CRP could also predict the short-term postoperative infectious complications as well as postoperative mortality (8, 9). The neutrophil-to-lymphocyte ratio (NLR) is a novel inflammatory marker and has been found to be associated with incidence, morbidity, and mortality in several systemic inflammatory diseases (10). It can be used as a prognostic predictor for metabolic syndrome in healthy population (11), diabetes in morbid obesity patients (12) and 30-day outcomes in bariatric surgery patients (13). The aim of the present study was to investigate the changes of NLR and CRP following bariatric surgery and to explore correlation between them and subclinical left ventricular myocardial function as well as the related factors including gender.

## Materials and methods

### Patient population

We retrospectively reviewed patients undergoing LSG in our hospital from 2017 to 2020 through the electronic medical record database. The inclusion criteria were: (1) patients with complete clinical information and laboratory data before and with follow up for around 6 months after surgery; (2) patients underwent standard echocardiography and 2D strain echocardiography before surgery. Patients with acute or chronic infection, chronic systemic inflammatory disease, secondary hypertension, acute stress, organs' failure and those who were receiving the medications affecting leukocytes were excluded.

### Variables collection

We recorded baseline patient characteristics including sex, age, height (cm), weight (kg), body mass index (BMI, kg/m^2^), history of hypertension, diabetes and dyslipidemia, respectively. Biochemical measurements included leukocyte count, neutrophil count, lymphocyte count, C-reactive protein (CRP), alanine amino transferase (ALT), aspartate amino transferase (AST), serum creatinine (Scr), uric acid (UA), glucose, insulin, C peptide, cholesterol, triglycerides, low density lipoprotein cholesterol (LDL-C) and high density lipoprotein cholesterol (HDL-C). All above were measured in the venous blood samples obtained in the morning after an 8-hour fasting. NLR was calculated by dividing the neutrophil count by the lymphocyte count. The average of NLR was around 2.0 in 20–30 years old (14).

### Echocardiography

Transthoracic echocardiograms were performed with a frame rate of 85 Hz. Standard echocardiographic parameters, including left ventricular (LV) dimension and LV ejection fraction (LVEF), peak early (*E*) and late (*A*) mitral inflow velocities, deceleration time of early mitral flow velocity (DT), early mitral annular velocity at the septal annular site (e1′) and at the lateral annular site (e2′). *E*/*E*′ = *E*/((e1′ + e2′)/2). LV mass was calculated with the following formula: LV mass (g) = 0.8 × {1.04 × ((LVID + PWT + SWT)^3^ − (LVID)^3^)} + 0.6. LV internal dimension (LVID), septal (SWT) and posterior wall thickness (PWT) were measured at end diastole according to American Society of Echocardiography recommendations (15). LVMI was corrected for the body surface. In addition, LV myocardial global longitudinal strain (LV-GLS) was measured by 2D speckle tracking (Vivid 7, GE Healthcare) which through the software semi-automatically tracked the myocardial motion during the cardiac cycle. LV-GLS was averaged from apical 4-, 2- and 3-chamber views. Abnormal myocardial function was defined as LV-GLS < 18% (16). The myocardial performance index (MPI) was measured by flow Doppler images (17). The cardiac time intervals included the isovolumic contraction time (IVCT), isovolumic relaxation time (IVRT), and ejection time (ET). MPI was defined as ((IVCT + IVRT)/ET).

### Statistical analysis

Date analysis was performed using SPSS 23.0 statistical software. Continuous variables were expressed as mean ± standard deviation (normal distribution, mean ± SD) or as median (nonparametric distribution, P25, P75). Categorical variables were expressed as numbers and percentages. We checked skewness and kurtosis for normality of our data. We assessed the changes of the parameters after bariatric surgery using a paired t test for normally distributed datasets or a Wilcoxon signed- rank test for non-normally distributed datasets where applicable. Univariate and multivariate logistic analysis were performed for MPI and *E*/*E*′, as well as NLR/CRP changes from baseline. Risk associations were expressed as Odds Ratio (OR) (95% CI). For all tests, a *p* value <0.05 was considered to be significant. Delta values were obtained by subtracting value measured 6 months after LSG from the value at baseline.

## Results

78 patients undergoing LSG met the inclusion criteria, 6 cases were excluded due to uninterpretable echocardiographic data. The follow-up interval was 6.7 ± 1.7months. Thus, totally 72 patients were evaluated in the final study with mean age of 29 ± 9.43 years (Table 1). 47% of all patients were male (*n* = 34). Average BMI was 39.6 ± 6.30 kg/m^2^, which was higher among males (41.5 ± 6.27 vs. 37.8 ± 5.86 kg/m^2^, *p* = 0.02). Preoperative comorbidity included hypertension in 18 of the patients, diabetes in 19 and dyslipidemia in 38. Patients with hypertension or diabetes had a higher CRP level (6.95 ± 2.89 vs. 5.27 ± 3.2 mg/L, *p* = 0.05; 6.83 ± 2.85 vs. 5.28 ± 3.2 mg/L, *p* = 0.05, respectively).

**Table 1:** Demographic characteristics and echocardiographic variables of study population before surgery.

	All (*n* = 72)	Male (*n* = 34)	Female (*n* = 38)
Age (years)	29.3 ± 9.43	28.3 ± 8.00	30.0 ± 10.59
BMI (kg/m^2^)	39.6 ± 6.30	41.5 ± 6.27	37.8 ± 5.86^*^
Obesity duration (years)	13.7 ± 9.09	14.7 ± 9.67	12.5 ± 8.26
Hypertension, *n* (%)	18 (25.0%)	8 (11.2%)	10 (13.8%)
Diabetes, *n* (%)	19 (26.3%)	9 (12.5%)	10 (13.8%)
Dyslipidemia, *n* (%)	38 (52.8%)	16 (22.2%)	22 (30.6%)
NLR	2.4 ± 1.59	2.21 ± 1.23	2.62 ± 1.84^*^
CRP (mg/L)	5.6 ± 3.17	5.78 ± 3.1	5.61 ± 3.31
AO (mm)	32.1 ± 2.88	33.9 ± 2.86	30.5 ± 1.78^**^
LA (mm)	39.3 ± 4.18	40.3 ± 4.50	38.3 ± 3.68
LVIDd (mm)	49.0 ± 3.63	50.6 ± 3.68	47.6 ± 2.96^**^
LVIDs (mm)	30.9 ± 2.64	31.8 ± 2.83	30.1 ± 2.20^**^
IVSd (mm)	10.3 ± 1.38	10.9 ± 1.41	9.8 ± 1.17^**^
LVPWd (mm)	9.9 ± 1.24	10.5 ± 1.28	9.4 ± 0.95^**^
LVMI (g/m^2^)	75.8 ± 14.47	78.4 ± 14.14	73.4 ± 14.53
LVEF (%)	66.8 ± 2.77	66.7 ± 3.02	66.8 ± 2.57
E wave (cm/s)	70.9 ± 11.65	67.3 ± 12.15	74.1 ± 10.31^**^
A wave (cm/s)	57.9 ± 15.36	56.9 ± 13.67	58.9 ± 16.86
DT (ms)	202.1 ± 29.70	199.1 ± 30.35	204.7 ± 29.25
*E*/*A*	1.3 ± 0.36	1.2 ± 0.30	1.3 ± 0.40
*E*/*E*′	6.6 ± 1.61	6.5 ± 1.89	6.7 ± 1.33
LV-GLS (%)	16.69 ± 3.04	15.62 ± 2.82	17.65 ± 2.94^*^
LV-MPI	0.31 ± 0.06	0.32 ± 0.06	0.30 ± 0.05

Presented as mean ± SD or *n* (%). Chi-square test for categorical data; Wilcoxon test for continuous variables.

* *p* < 0.05 vs. male group.

** *p* < 0.01 vs. male group. *p*-values < 0.05 was considered significant.

BMI, body mass index; NLR, neutrophil-to-lymphocyte ratio; CRP, C-reactive protein; AO, Aortic root diameter; LA, Left atrium diameter; LVIDd, left ventricular internal diameter at end-diastole; LVIDs, left ventricular internal diameter at end-systole; IVSd, diastolic inter-ventricular septum thickness; LVPWd, diastole left ventricular posterior wall thickness; LVMI, LV mass index; LVEF, LV ejection fraction; DT, deceleration time; LV-GLS, left ventricular global longitudinal strain; LV-MPI, left ventricular myocardial performance index.

**Figure 1:**
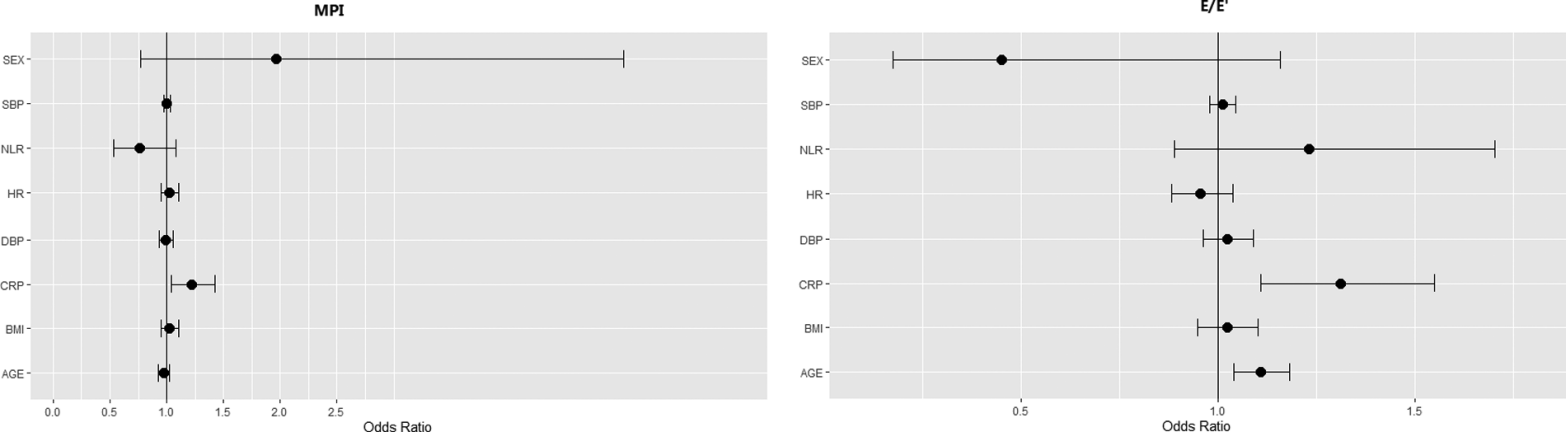
Effect of clinical parameters and inflammatory factors on MPI and *E*/*E*′: results of univariate analysis. In univariate logistic regression models, baseline MPI and *E*/*E*′ were used as the outcome and medians of the outcomes (MPI > 0.305 (*n* = 36), *E*/*E*′ > 6.5 (*n* = 35)) were used as the events. Risk associations were expressed as Odds Ratio (OR) (95% CI). *p*-values < 0.05 was considered significant. SBP, systolic blood pressure; NLR, neutrophil-to-lymphocyte ratio; HR, heart rate; DBP, diastolic blood pressure; CRP, C-reactive protein; BMI, body mass index.

Echocardiographic indices were also shown in Table 1. All patients had normal LVEF prior to LSG. Median LVIDd and LVMI were 49.0 ± 3.63 mm and 75.8 ± 14.47 g/m^2^, respectively. 17 patients were found with MPI > 0.34 and 48 patients were found with decreased GLS, indicating subclinical impaired LV function. Male patients had a larger LV diameter, thicker LV walls and lower LV-GLS than the females (all *p* < 0.05). In logistic regression models, those patients with higher CRP were more likely to have higher MPI (OR = 1.218 (95%CI 1.040, 1.426); *p* = 0.0142) and higher *E*/*E*′ (OR = 1.364 (95%CI 1.124, 1.655); *p* = 0.0017) (shown in Figure 1, Supplementary Tables S1, S2).

The pre- and postoperative evaluation of biochemical measurements were summarized in Table 2. After LSG, all patients managed to achieve significant reduction on BMI (39.6 ± 6.30 vs. 28.9 ± 6.44 kg/m^2^, *p* < 0.0001). The blood pressure and heart rate got significantly lower. NLR and CRP had both significantly decreased (NLR: 2.4 ± 1.59 vs. 1.7 ± 0.86, CRP: 5.6 ± 3.17 vs. 2.1 ± 2.35 mg/L, respective, both *p* < 0.0001). There were also notable reductions in ALT, AST, UA, glucose, insulin, C peptide (all *p* < 0.0001). Moreover, LSG could significantly improve the lipid profile. Cholesterol, triglycerides and LDL-C significantly decreased while HDL-C significantly increased (all *p* < 0.05). The univariate analysis demonstrated that female might gain more reduction in NLR than male (OR = 2.81 (95%CI 1.078, 7.327); *p* = 0.0345). Adjusting for age, delta BMI and prevalence of hypertension, the result was similar (OR = 3.14 (95%CI 1.112, 8.870); *p* = 0.031) (shown in Table 3, 4). Unfortunately, there were no statistical correlations between the reduction of inflammatory marks and delta BMI, delta SBP, delta TC, delta TG and delta LDL-C. Only delta HDL was shown to be significantly correlated with CRP decrease in multivariate logistic regression analysis. (OR 0.086, (95%CI 0.008, 0.96); p = 0.046)).

**Table 2:** Comparison of various measured parameters before and after bariatric surgery.

	Pre-operation	Post-operation	*p* value
BMI (kg/m^2^)	39.6 ± 6.30	28.9 ± 6.44	<0.0001
SBP (mmHg)	132.2 ± 14.49	122.3 ± 11.99	<0.0001
DBP (mmHg)	83.4 ± 7.60	77.8 ± 8.89	<0.0001
HR (bpm)	79.4 ± 6.42	76.5 ± 6.40	<0.0001
NLR	2.4 ± 1.59	1.7 ± 0.86	<0.0001
CRP (mg/L)	5.6 ± 3.17	2.1 ± 2.35	<0.0001
ALT (U/L)	65.6 ± 45.78	14.7 ± 9.02	<0.0001
AST (U/L)	34.5 ± 19.99	17.7 ± 15.86	<0.0001
Scr (µmol/L)	58.6 ± 12.56	60.8 ± 12.25	0.006
UA (mmol/L)	0.5 ± 0.65	0.5 ± 0.97	<0.0001
Glucose (mmol/L)	6.9 ± 3.37	4.8 ± 0.70	<0.0001
Insulin (pmol/L)	33.3 ± 29.98	11.4 ± 7.58	<0.0001
C peptide (μg/L)	3.7 ± 2.06	2.4 ± 1.08	<0.0001
TC (mmol/L)	4.9 ± 1.07	4.5 ± 0.88	0.0015
TG (mmol/L)	2.6 ± 4.11	1.1 ± 0.69	<0.0001
LDL-C (mmol/L)	3.1 ± 0.86	2.9 ± 0.68	0.0065
HDL-C (mmol/L)	1.0 ± 0.19	1.2 ± 0.26	<0.0001

Presented as mean ± SD. Wilcoxon signed- rank test was used.

*p*-values <0.05 was considered significant.

BMI, body mass index; SBP, systolic blood pressure; DBP, diastolic blood pressure; NLR, neutrophil-to-lymphocyte ratio; CRP, C-reactive protein; ALT, alanine amino transferase; AST, aspartate amino transferase; Scr, serum creatinine; UA, uric acid; TC, cholesterol; TG, triglycerides; LDL-C, low density lipoprotein cholesterol; HDL-C, high density lipoprotein cholesterol.

**Table 3:** Univariate logistic regression analysis of the association of the association between delta NLR, delta CRP and the influencing factors.

Parameters	Delta NLR			Delta CRP		
	OR	95% CI	*p* value	OR	95% CI	*p* value
Age, years	0.976	0.928, 1.026	0.3460	1.019	0.969, 1.071	0.4596
Male, *n* (%)	2.811	1.078, 7.327	0.0345	1.565	0.617, 3.971	0.3460
Hypertension = yes, *n* (%)	1.41	0.520, 3.860	0.505	1.667	0.524, 5.298	0.3866
Diabetes = yes, *n* (%)	1.428	0.496, 4.113	0.5094	0.483	0.164, 1.418	0.1854
Dyslipidemia = yes, *n* (%)	2.190	0.853, 5.625	0.1032	0.800	0.317, 2.022	0.6374
Delta BMI, kg/m^2^	0.963	0.883, 1.051	0.3985	1.040	0.952, 1.137	0.3818
Delta SBP, mmHg	0.977	0.948, 1.008	0.1386	1.025	0.994, 1.056	0.1217
Delta DBP, mmHg	0.979	0.929, 1.032	0.4337	1.013	0.962, 1.067	0.6269
Delta HR, beat/min	0.985	0.929, 1.045	0.6238	1.076	0.997, 1.160	0.0603
Delta TG mmol/L	1.033	0.896, 1.192	0.6518	0.952	0.813, 1.114	0.5373
Delta TC mmol/L	0.949	0.616, 1.463	0.8141	0.643	0.370, 1.118	0.1176
Delta LDL mmol/L	0.710	0.377, 1.336	0.2883	0.916	0.500, 1.678	0.7767
Delta HDL mmol/L	1.398	0.172, 11.353	0.7536	0.085	0.008, 0.859	0.0368

Univariate logistic analysis were performed for NLR and CRP changes from baseline. Median NLR change (>−0.5, *n* = 37) and median CRP Change (>−3, *n* = 36) were used as the event in the model.

*p*-values < 0.05 was considered significant.

NLR: neutrophil-to-lymphocyte ratio; CRP, C-reactive protein; OR: odds ratio; BMI, body mass index; SBP, systolic blood pressure; DBP, diastolic blood pressure; TC, cholesterol; TG, triglycerides; LDL-C, low density lipoprotein cholesterol; HDL-C, high density lipoprotein cholesterol. Delta parameters meant the changes from baseline.

**Table 4:** Association between delta NLR and the related factors including gender using multivariate logistic analysis.

Parameters	Multivariate model		
	OR	95% CI	*p* value
Age, years	0.97	0.92, 1.02	0.327
Male, *n* (%)	3.14	1.11, 8.87	0.031
Delta BMI, kg/m^2^	0.95	0.87, 1.04	0.271
Hypertension = yes, *n* (%)	1.57	0.51, 4.86	0.431

Risk associations were expressed as Odds Ratio (OR) (95% CI).

*p*-values < 0.05 was considered significant.

BMI: body mass index; SBP, systolic blood pressure; NLR: neutrophil-to-lymphocyte ratio; OR: odds ratio.

## Discussion

The present study confirmed that inflammation played a critical role in severe obesity. A great proportion of individuals with preserved LV ejection fraction had abnormal subclinical myocardial function as shown by increased MPI and decreased GLS. We indicated, for the first time, a significant correlation between MPI and CRP among obese patients. Furthermore, at 6 months post-surgery, the majority of individuals' inflammatory burden significantly improved, which was in agreement with previous studies (6). Female patients might benefit more from bariatric surgery on subclinical inflammation, that was first reported, to the best of our knowledge.

In the current study, a high proportion of patients were found with elevated NLR (44.4%) and CRP (75%) before surgery, which was consistent with an underlying inflammatory process (4). Similar to the findings of Bulur O et al. and Randell EW et al., we also found that the levels of NLR and CRP significantly reduced after LSG surgery (6, 18). As non-specific inflammatory markers, those reductions indicated the improvement of systemic inflammatory response. It may be correlated with the significant reduction of adipose tissue inflammation (19), macrophage infiltration (20) and insulin resistance (21), which could partly explain the surgery-induced remission of diabetes and hypertension (22, 23). It may also be interrelated with the resolution of nonalcoholic fatty liver disease (24). Our study confirmed many benefits of LSG towards weight loss. Blood pressure, hepatic function, glucose and all the lipid profile significantly improved after LSG. Those indicated that the role of LSG on inflammatory burden involved multiple tissues and organs.

A novel finding of our study was that the bariatric surgery-induced benefits on inflammation was gender-related. Although there were higher levels of BMI, ALT, AST as well as LV diameter and LV mass in male patients, females gained a greater reduction after surgery. This indicated that the improvement of the inflammatory state was greater in females than in males. It might contribute to the fact that the overall mortality rate and the 30-day mortality were found significantly lower in females compared to males (25). However, the mechanisms underlying the gender-related difference have not yet been clearly elucidated. As we all know, the distribution of adipose tissue differed by gender. Females tended to accrue more fat in subcutaneous prior to menopause, while males accrued more fat in visceral (26). Studies reported that bariatric surgery as well as other weight loss strategies leaded to greater loss of adipose tissue in subcutaneous than in visceral (27). The reduction of adipose tissue could decrease the secretion of inflammatory adipokines and lower the infiltration of bone marrow-derived immune cells which produce cytokines and chemokines (28). Besides, sex hormones might be one possible factor influencing inflammation (29). Before menopause, females had a stronger inflammatory response with a significant peak level of NLR in their 30s (14, 30). Bariatric surgery could mediate sex hormones (31), and thus improve the regression of polycystic ovary syndrome and promote successful pregnancy (32), which might be related to the additional reduction of inflammatory burden in females. Certainly, there was still much to learn about the role of gender and sex hormones on inflammation (33).

MPI was a reliable and reproducible doppler-derived index that reflected both LV systolic and diastolic function, which was first introduced by Tei in 1995 (17). GLS, measured by speckle tracking echocardiography, was a novel parameter to assess the subtle change in LV systolic function (34). The present study indicated, for the first time, a significant correlation between MPI, *E*/*E*′ and CRP among obese patients. Whereas no association was found between decreased LV-GLS and inflammatory markers. This result indicated that obesity-induced inflammation, which was known as a persistent and low-grade inflammatory response, was related to myocardial dysfunction, especially the diastolic dysfunction. Besides, there was accumulating evidence indicating the correlation between increased epicardial adipose tissue (EAT) and LV diastolic dysfunction (35, 36). EAT could not only mediate and induce the adverse consequences of obesity or systemic inflammation on the heart, but also was a metabolic activator which could secrete some proinflammatory adipokines resulting in atrial and ventricular fibrosis (37). Myocardial reactive fibrosis provoked by chronic inflammation was the key factor for diastolic dysfunction (38). Recent studies demonstrated that CRP was not only a inflammation marker, but also a direct participant in inflammation (39). Bock C et al. found that CRP could modulate the intracellular calcium concentration with dose-dependent (40). Cytosolic and mitochondrial calcium handling was a significant contributor to myocardial function (41). However, further research is needed to identify the predictive inflammatory markers associated with the improvement of cardiac function post-surgery.

This study inevitably had some limitations. A major limitation is its retrospective design which could not allow us to draw causal relationship between inflammation markers and cardiac function in obesity. And this was a single-center study with small sample size. Those may limit the interpretation of our results, though an effort was made to include as many perioperative variables as possible and extensively adjust for confounding factors. Only a small proportion of patients underwent echocardiography at follow-up, which couldn't be included in the dataset. Therefore, multicenter studies with larger sample size are needed to further observe and confirm the dynamic changes of inflammation markers and cardiac function.

## Conclusions

The present study indicated a significant correlation between subclinical cardiac dysfunction and CRP among obese patients. Bariatric surgery was effective to improve the inflammation state, particularly to reduce the level of NLR among female patients.

## Data Availability

The original contributions presented in the study are included in the article/**Supplementary Material**, further inquiries can be directed to the corresponding author/s.
