# Supplementary material for: Might female patients benefit more from bariatric surgery with respect to inflammation
Source: Front Surg. 2022 Aug 8;9:890116. doi: 10.3389/fsurg.2022.890116 (PMC9393378; doi:10.3389/fsurg.2022.890116)
Supplement: Supplementary file 1 [file Data_Sheet_1_v1.pdf]

Supplementary Table 1 Effect of clinical parameters and inflammatory factors on MPI and E/E’: results of univariate analysis

Parameters	MPI				E/E’		
	OR	95%CI	P value		OR	95%CI	P value
**Age, years**	0.974	0.926, 1.025	0.312		1.109	1.040, 1.182	0.002
**Male, n(%)**	1.964	0.768, 5.022	0.159		0.450	0.175, 1.158	0.098
**BMI (kg/m^2)**	1.024	0.950, 1.103	0.536		1.023	0.949, 1.101	0.555
**SBP (mmHg)**	1.003	0.971, 1.036	0.844		1.012	0.979, 1.045	0.482
**DBP (mmHg)**	0.993	0.934, 1.056	0.827		1.024	0.963, 1.090	0.448
**CRP (mg/L)**	1.218	1.040, 1.426	0.014		1.311	1.110, 1.549	0.014
**NLR**	0.762	0.536, 1.083	0.129		1.231	0.889, 1.704	0.211
**TG (mmol/L)**	0.976	0.865, 1.101	0.694		1.045	0.913, 1.196	0.520
**TC (mmol/L)**	1.176	0.756, 1.831	0.472		0.793	0.505, 1.245	0.314
**LDL (mmol/L)**	1.166	0.674, 2.015	0.583		0.713	0.405, 1.254	0.240
**HDL (mmol/L)**	0.624	0.054, 7.177	0.705		0.586	0.051, 6.762	0.668
**Diabetes=yes, n(%)**	1.154	0.404, 3.295	0.789		3.052	1.005, 9.271	0.049
**Dyslipidemia=yes, n(%)**	1.250	0.495, 3.157	0.637		1.125	0.446, 2.840	0.803

In univariate logistic regression models, baseline MPI and E/E' were used as the outcome and medians of the outcomes (MPI >0.305(n=36), E/E’ > 6.5(n=35)) were used as the events. Risk associations were expressed as Odds Ratio (OR) (95% CI).

p-values < 0.05 was considered significant.

SBP, systolic blood pressure; NLR, neutrophil-to-lymphocyte ratio; HR, heart rate; DBP, diastolic blood pressure; CRP, C- reactive protein; BMI, body mass index; TC, cholesterol; TG, triglycerides; LDL-C, low density lipoprotein cholesterol; HDL-C, high density lipoprotein cholesterol.

Supplementary Table 2 Association between clinical parameters, inflammatory factors and E/E'

**Parameters**	**Multivariate model**		
	**OR**	**95% CI**	**P value**
**Age, years**	1.121	1.040-1.209	0.0030
**Male, n(%)**	0.381	0.119-1.219	0.1038
**CRP, mg/L**	1.364	1.124-1.655	0.0017
**Diabetes=yes, n(%)**	1.872	0.504-6.952	0.3487

Those with a *p* value <0.1 in univariate analyses were included in the multivariate model. Risk associations were expressed as Odds Ratio (OR) (95% CI).

p-values < 0.05 was considered significant.

CRP, C-reactive protein
